# Autophagy regulates trophoblast invasion by targeting NF-κB activity

**DOI:** 10.1038/s41598-020-70959-2

**Published:** 2020-08-20

**Authors:** Soo-young Oh, Jae Ryoung Hwang, Minji Choi, Yoo-Min Kim, Jung-Sun Kim, Yeon-Lim Suh, Suk-Joo Choi, Cheong-Rae Roh

**Affiliations:** 1 Department of Obstetrics and Gynecology, Samsung Medical Center, Sungkyunkwan University School of Medicine, Seoul, Republic of Korea; 2 Sungkyunkwan University School of Medicine, Samsung Biomedical Research Institute, Samsung Medical Center, Seoul, Republic of Korea; 3 Department of Health Sciences and Technology, SAIHST, Sungkyunkwan University, Seoul, Republic of Korea; 4 Department of Obstetrics and Gynecology, Chung-Ang University Hospital, Chung-Ang University College of Medicine, Seoul, Republic of Korea; 5 Department of Pathology, Samsung Medical Center, Sungkyunkwan University School of Medicine, Seoul, Republic of Korea

**Keywords:** Macroautophagy, Translational research

## Abstract

Preeclampsia is one of the most serious complications of pregnancy, affecting 5–10% of parturients worldwide. Recent studies have suggested that autophagy is involved in trophoblast invasion and may be associated with defective placentation underlying preeclampsia. We thus aimed to understand the mechanistic link between autophagy and trophoblast invasion. Using the two most commonly used trophoblast cell lines, JEG-3 and HTR-8/SVneo, we inhibited autophagy by ATG5 and beclin-1 shRNA. Conversion of LC3-II was evaluated in ATG5 and beclin-1 knock-down cells in the presence of the lysosomal protease inhibitors E-64d and pepstatin A, to detect the efficiency of autophagy inhibition. Upon autophagy inhibition, we measured cell invasion, activity of NF-κB and related signaling pathways, MMP-2, MMP-9, sFlt-1, and TNF-α levels. Autophagy inhibition increased the invasiveness of these trophoblastic cell lines and increased Akt and NF-κB activity as well as p65 expression. Of note, an NF-κB inhibitor significantly attenuated the trophoblast invasion induced by autophagy inhibition. Autophagy inhibition was also associated with increased MMP-2 and MMP-9 levels and decreased the production of sFlt-1 and TNF-α. Collectively, our results indicate that autophagy regulates trophoblast invasiveness in which the NF-κB pathway and MMP-2, MMP-9, sFlt-1 and TNF-α levels are affected.

## Introduction

Abnormal shallow placentation is implicated in the pathophysiology of preeclampsia, one of the most serious complications of pregnancy that accounts for considerable maternal and neonatal mortality and morbidity^1^. Normal placentation requires formation of the anchoring villi, proliferation of cell columns, and invasion of extravillous trophoblasts (EVTs) into the maternal decidua, myometrium, and spiral arteries, where vascular alterations enable adequate blood supply to the growing fetus^2^. The process of EVT invasion into the maternal tissue requires the attachment of cytotrophoblast cell columns to the decidua, along with proteolytic degradation of the extracellular matrix and trophoblast cell migration from the decidua to the maternal myometrium, forming placental beds. However, in pregnancies complicated by preeclampsia, cytotrophoblasts fail to differentiate along the invasive pathway and undergo widespread apoptosis, leading to limited invasion into the uterus^3^.

Upon stresses such as hypoxic conditions or nutrition deficiency, autophagy is activated. The ULK1/ATG13/ATG101 complex initiates phagophore formation, also called the isolation membrane, through activation of Beclin-1. Beclin-1 is an orthologue of the yeast autophagy-related gene 6 (Atg6) and plays a central role in the phagophore formation via the interaction with other proteins such as lipid kinase Vps34^4^. A complex of ATG12/ATG5/ATG16L1 induces conjugation of phosphatidylethanolamine (PE) in LC3 and an autophagosome is subsequently formed. Thus, PE-conjugated LC3, called LC3-II, is used as an autophagy marker protein. Autophagosomes dock to the cargo protein p62/SQSTM1 and fuse with lysosomes which are called autolysosomes, and finally the autophagosomes are degraded^5^.

Autophagy is understood as a mechanism for both cell death and cytoprotection depending on the cellular types and contexts, and it has been implicated in various physiological and pathological conditions^6,7^. Without exception, autophagy is also involved in numerous physiological and pathological processes of human reproduction and pregnancy complications^8–11^. Undue autophagic activity upon non-physiological stimuli such as excessive inflammation or severe oxidative stress may limit trophoblast invasion, resulting in defective placentation^12^. Recent studies have revealed increased autophagy in placentas from pregnancies complicated by preeclampsia or fetal growth restriction^13–17^. Moreover, the severity of preeclampsia or fetal growth restriction is positively correlated with enhanced autophagosome formation, suggesting that altered autophagic activity is one of the mechanisms that underlie defective trophoblast invasion^1,18^. In contrast, impaired autophagy has also been implicated in the pathophysiology of preeclampsia. According to a study by Nakashima et al., soluble endoglin, an anti-angiogenic factor, inhibits autophagy in EVTs under hypoxia, resulting poor invasion and vascular remodeling^19^. These authors also demonstrated that the expression of p62/SQSTM1, a hallmark of autophagic inhibition, was significantly increased in EVTs in placental bed biopsies from preeclampsia patients compared to normal pregnancies.

NF-κB, a nuclear transcriptional regulator, is involved in various cellular functions and plays a pivotal role in innate and adaptive immune responses^20^. p65, a subunit of NF-κB, is degraded by autophagy. For example, the toll-like receptor 2 signal can induce the ubiquitination of p65, which is subsequently degraded by p62/SQSTM1-mediated autophagy^21^. Knockdown of *ATG3-* or *ATG5*-activated NF-κB has also been reported to inhibit autophagy^22^. On the contrary, autophagy inhibition and p62 accumulation were found to inhibit NF-kB activity^23^. Collectively, many modes of crosstalk occur between autophagy and NF-κB.

The objective of this study was to understand the link between autophagy and trophoblast invasion and elucidate the underlying molecular mechanism. To achieve this goal, we investigated the invasiveness of trophoblastic cells after suppressing autophagy using the two most commonly used trophoblast cell lines, JEG-3 cells (choriocarcinoma cell line) and HTR-8/SVneo cells (first trimester EVT cell line). We report here that inhibiting autophagy significantly increased trophoblast invasiveness, in conjunction with enhanced expression of p65, a subunit of NF-κB and NF-κB activity, as well as increased matrix metalloproteinase (MMP)-2 and MMP-9 levels, while decreasing the production of soluble fms-like tyrosine kinase-1 (sFlt-1) and tumor necrosis factor-α (TNF-α). Conversely, inhibiting NF-κB activity significantly alleviated the increased trophoblast invasion that occurred upon autophagy suppression. Taken together, we show that inhibition of autophagy enhances trophoblast cell invasion, through the involvement of activation of NF-κB related signaling pathways and changes in the secretion of MMP-2, MMP-9, sFlt-1, and TNF-α.

## Results

### Autophagy inhibition increases trophoblast invasion

**Figure 1:**
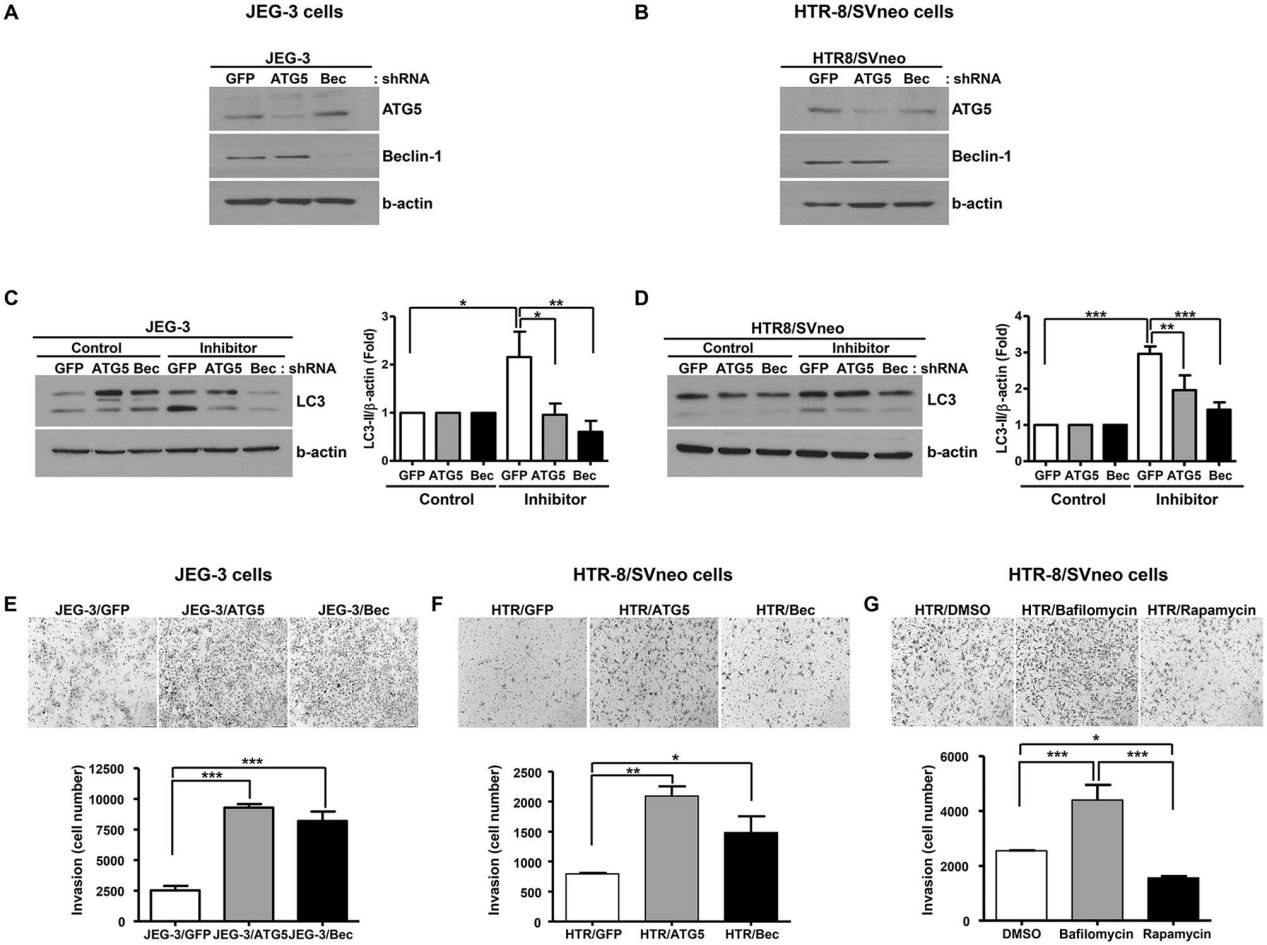
Effects of autophagy inhibition on the invasive potential of JEG-3 and HTR-8/SVneo cells upon ATG5 or beclin-1 knockdown. (**A**,**B**) Western blot for ATG5 and beclin-1 upon treatment of ATG5 or beclin-1 shRNA in JEG-3 and HTR-8/SVneo cells. (**C**,**D**) Flux assay with a lysosomal protease inhibitor mixture, E-64d and pepstatin A, in JEG-3 and HTR-8/SVneo cells. Cells were treated with either the mixture of E-64d and pepstatin A or the vehicle, for 4 h. Three different experiments were performed and representative blots are shown with densitometric analysis for these experiments. The amount of LC3-II normalized to the amount of β-actin is represented by a bar graph (*p < 0.05, **p < 0.01, ***p < 0.001). (**E**,**F**) Invasion assays performed with JEG-3 and HTR-8/SVneo cells. Cells invading the Matrigel were stained with hematoxylin/eosin and counted under a microscope. Results of the invasion assay are represented in a bar graph generated with data obtained from three different experiments (n = 3, *p < 0.05; lower panel) and representative pictures from three different assays are shown in the upper panel.

To determine the role of autophagy in trophoblastic cell invasion, we first inhibited autophagy by stably transfecting JEG-3 cells and HTR-8/SVneo cells with shRNA against ATG5 (autophagy regulated 5) or beclin-1, which are among common target for autophagy inhibition in mammalian autophagy research^24^. We confirmed that ATG5 and beclin-1 expression were almost completely silenced in both cell lines (Fig. 1A,B). To validate the inhibition of autophagic flux by ATG5 and beclin-1 shRNAs, we treated cells with a lysosomal inhibitor mixture of E-64d and pepstatin A. As shown in Fig. 1C,D, we confirmed that LC3-II expression in the presence of lysosomal inhibitors was increased in control cells but not in ATG5 and beclin-1 shRNA-infected cells, indicating impaired autophagy. We also determined the efficiency of autophagy inhibition upon treatment of rapamycin in both cell lines. In JEG-3 cells, increases in expression of LC3-II by rapamycin was modest in the control, ATG5 and beclin-1 knock-down cells and the p-values were not statistically significant compared with rapamycin-untreated cells (Supplementary Fig. S1A). However, in control HTR-8/GFP cells, rapamycin markedly increased expression of LC3-II, which was significantly decreased in ATG5 and beclin-1 knock-down cells (Supplementary Fig. S1B). Collectively, these results demonstrate that the shRNAs for ATG5 and beclin-1 efficiently inhibit autophagy flux in both trophoblast cell lines.

An invasion study using Matrigel assays revealed that autophagy inhibition by ATG5 or beclin-1 shRNA caused a significant increase in the invasiveness of JEG-3 cells (Fig. 1E). Similarly, inhibition of autophagy in HTR-8/SVneo cells also manifested an increase in invasiveness compared to cells transfected with control shRNA (Fig. 1F). In HTR-8/SVneo cells, we also confirmed that pharmacologic treatment of bafilomycin (autophagy inhibitor) increased trophoblast invasion, whereas rapamycin (autophagy inducer) treatment significantly decreased trophoblast invasion (Fig. 1G).

### Autophagy inhibition enhances NF-κB activity and increases MMP-2 and MMP-9 levels

**Figure 2:**
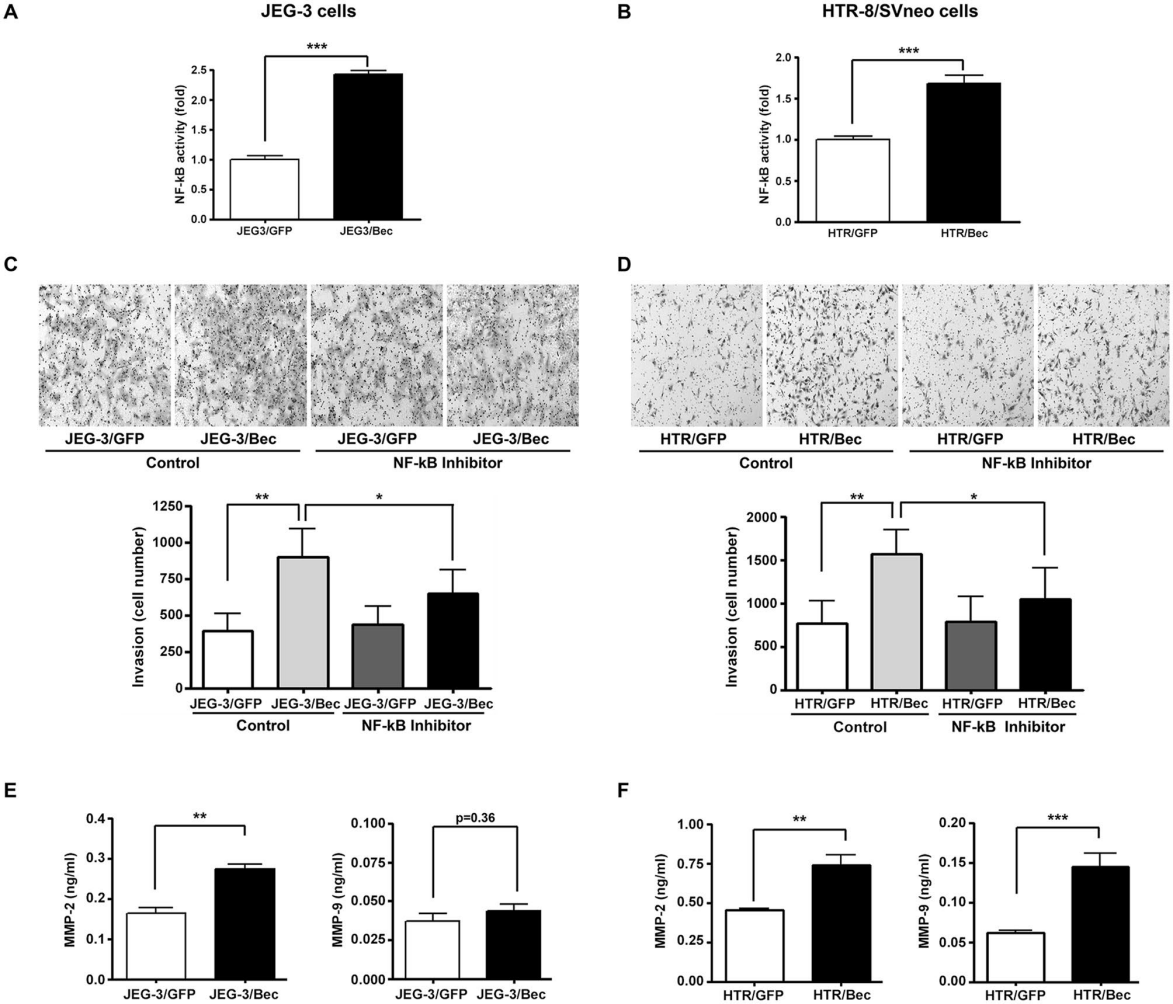
Effects of autophagy inhibition by beclin-1 knockdown on NF-κB activity and MMP-2 and MMP-9 levels in JEG-3 and HTR-8/SVneo cells. (**A**,**B**) NF-κB activity upon treatment of beclin-1 shRNA in JEG-3 and HTR-8/SVneo cells. Cells expressing only GFP and beclin-1 shRNA were plated on 24-well plates and transfected with plasmids encoding NF-κB-Luc and pRL-SV40. Luciferase activity was measured using a dual luciferase reporter assay kit. Results are shown as the mean ± SEM of duplicate observations from three different experiments (n = 6, ***p < 0.001). (**C**,**D**) Invasion assays were performed with JEG-3 and HTR-8/SVneo cells in the presence of either an NF-κB inhibitor or control peptide. Invasive cells were stained with hematoxylin/eosin and counted under a microscope. Results of the invasion assay are represented in a bar graph generated with data obtained from three different experiments (n = 3, *p < 0.05, **p < 0.01) and representative pictures from three different assays are shown in the upper panel. (**E**,**F**) MMP-2 and MMP-9 levels were measured by ELISA in the media obtained from JEG-3 and HTR-8/SVneo stable cells upon control and beclin-1 shRNA treatment. Results are shown as the mean ± SEM of triplicate observations from three different experiments (n = 3, **p < 0.01, ***p < 0.001).

To further study the signaling pathway involved in increased invasion by autophagy inhibition in JEG-3 cells and HTR-8/SVneo cells, we measured NF-κB activity after autophagy inhibition in both cell lines. As shown in Fig. 2A,B, we found that NF-κB activity was significantly enhanced after suppressing autophagy by beclin-1 shRNA in both cells, to levels approximately two times of the controls. To investigate the involvement of altered NF-κB activity in autophagy inhibition-mediated trophoblast invasion, we performed invasion assays of JEG-3 and HTR-8/SVneo stable cells in the presence of an NF-κB inhibitor. As shown in Fig. 2C,D, treatment with an NF-κB inhibitor significantly attenuated increased trophoblast invasion upon autophagy inhibition in both cells. Of note, NF-κB inhibition alone did not affect trophoblast invasion in the control. Since MMP-2 and MMP-9 are among the key players of trophoblast invasion, we measured MMP-2 and MMP-9 levels following autophagy inhibition. As shown in Fig. 2E, treatment with beclin-1 shRNA significantly increased MMP-2 levels in JEG-3 cells whereas the increase of MMP-9 was not statistically significant. However, in HTR-8/SVneo cells, both MMP-2 and MMP-9 levels were significantly increased compared to the control upon autophagy inhibition by beclin-1 shRNA (Fig. 2F).

### Autophagy inhibition increases the expression of p65 and phosphorylated and total Akt

**Figure 3:**
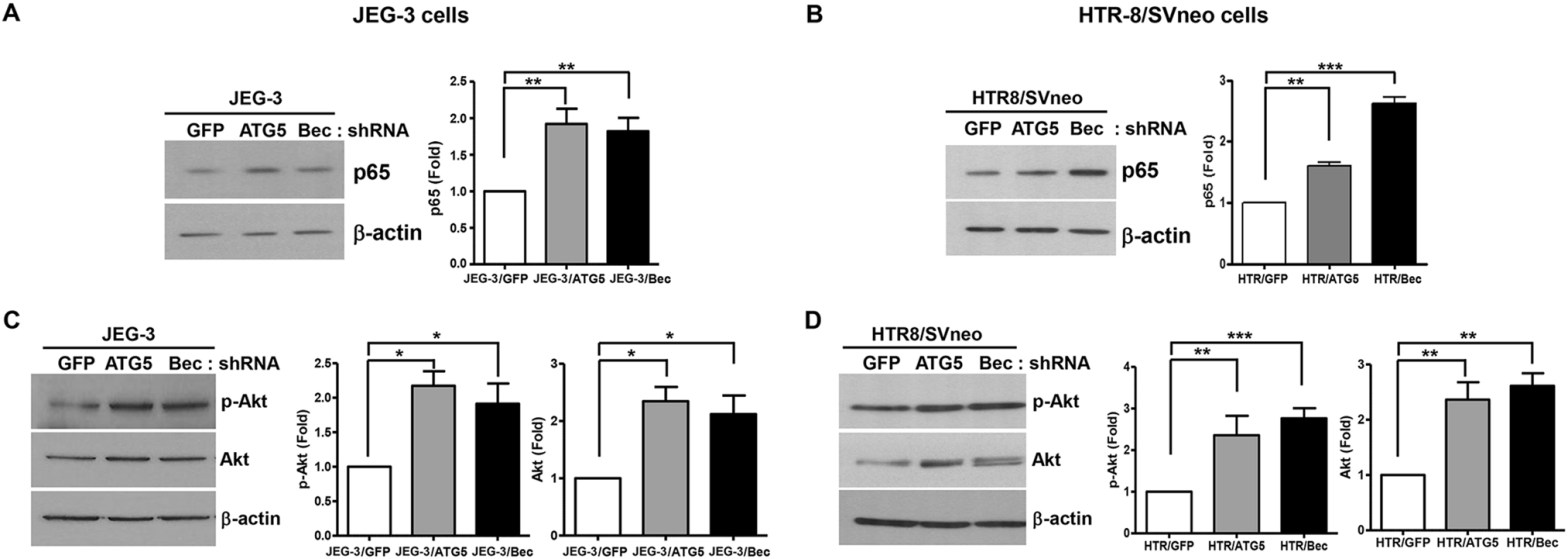
The expression of p65, phosphorylated Akt, and total Akt in autophagy-deficient JEG-3 and HTR-8/SVneo cells. (**A**,**B**) Western blot for p65, a subunit of NF-κB, in JEG-3 and HTR-8/SVneo cells expressing GFP or shRNA against ATG5 or beclin-1. The bar graph in the right panel represents p65 expression levels determined by densitometry, normalized to β-actin expression (n = 3, ***p < 0.001, **p < 0.01). (**C**,**D**) Phosphorylated Akt and total Akt were analyzed by Western blot using phospho-specific Akt antibody and anti-Akt antibody which recognizes both phosphorylated and unphosphorylated Akt. The bar graphs in the right panel represents phosphorylated and total Akt expression levels determined by densitometry, normalized to β-actin expression (n = 3, ***p < 0.001, **p < 0.01, *p < 0.05).

Next, we evaluated the expression of p65, an NF-κB subunit that is known to be degraded by autophagy^21^. As shown in Fig. 3A,B, inhibition of autophagy by knockdown of ATG5 or beclin-1 increased the level of p65 protein in JEG-3 cells and HTR-8/SVneo cells, respectively. In order to study the effect of autophagy inhibition on the upstream of the NF-κB signaling pathway, we analyzed the expression of Akt (total and phosphorylated), which has been reported as both an upstream and downstream target of NF-κB^25^, upon autophagy inhibition in both JEG-3 and HTR-8/SVneo cells. Inhibition of autophagy by ATG5 or beclin-1 shRNAs increased phosphorylated Akt (p-Akt) as well as total Akt compared with the control in JEG-3 and HTR-8/SVneo cells (Fig. 3C,D, respectively).

### Autophagy inhibition decreases the production of sFlt-1 and TNF-α

**Figure 4:**
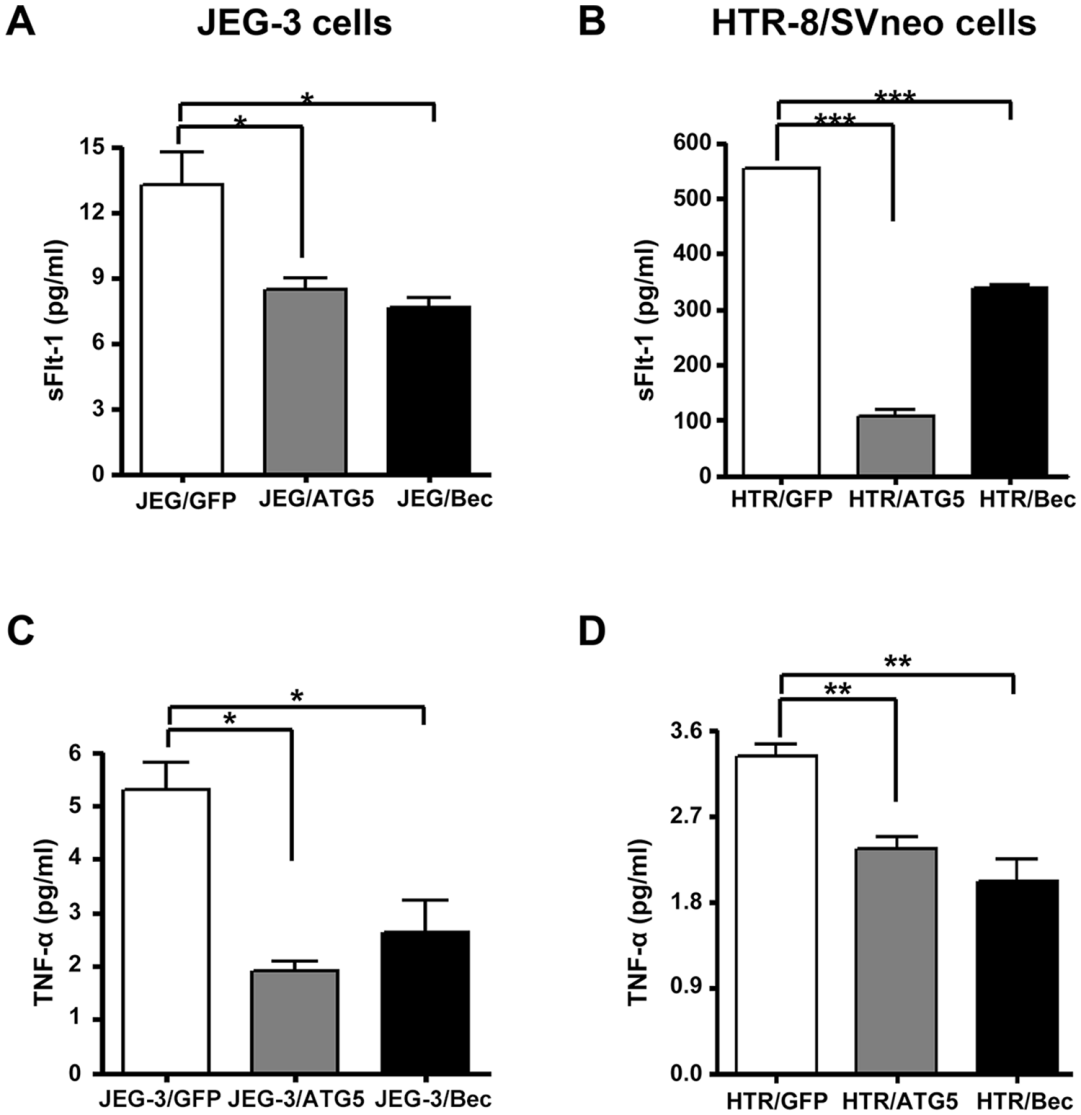
The levels of sFlt-1 and TNF-α in autophagy-deficient JEG-3 and HTR-8/SVneo cells. (**A**,**B**) sFlt-1 levels were measured by ELISA in the media obtained from JEG-3 and HTR-8/SVneo stable cells upon treatment of ATG5 or beclin-1 shRNA. Results are shown as the mean ± SEM of triplicate observations from three different experiments (n = 3, *p < 0.05, ***p < 0.001). (**C**,**D**) TNF-α levels were measured by ELISA in the media obtained from JEG-3 and HTR-8/SVneo stable cells upon treatment of ATG5 or beclin-1 shRNA in JEG-3 and HTR-8/SVneo cells. Results are shown as the mean ± SEM of triplicate observations from three different experiments (n = 3, *p < 0.05, **p < 0.01).

Since elevation of sFlt-1, one of the most important anti-angiogenetic factors in placental development, is associated with poor placentation^26^, we measured the secretion of sFlt-1 upon autophagy inhibition. As presented in Fig. 4A,B, the levels of sFlt-1 were significantly reduced in ATG5 and beclin-1 knockdown cells compared with the control in JEG-3 cells and HTR-8/SVneo cells. Of note, we found that the concentration of sFlt-1 in HTR-8/SVneo cells is exceedingly higher than that in JEG-3 cells, reflecting differences in the characteristics of these trophoblast cell lines. We also measured the levels of PlGF and VEGF-A upon ATG5 and beclin-1 knockdown in both cell lines (Supplementary Fig. S2A–D). In this case, we found that the response of JEG-3 cells and HTR-8/SVneo cells upon autophagy inhibition were different and the baseline levels of these angiogenic factors was also different between the two cell lines. The concentrations of PlGF and VEGF-A were increased in autophagy-inhibited JEG-3 cells compared with the control, while in contrast, in HTR-8/SVneo cells, PlGF and VEGF-A were all decreased in ATG5 and beclin-1 knockdown cells compared with control cells. We also assayed for TNF-α, which is an important inflammatory cytokine in the maternal–fetal interface in establishing pregnancy. As a result, we found that secreted TNF-α in JEG-3 and HTR-8/SVneo cells was decreased in autophagy-deficient conditions (in Fig. 4C,D, respectively). Regarding the levels of IL-6, we again found that secreted levels of IL-6 upon autophagy inhibition were different between the two cell lines, showing increased IL-6 in JEG-3 cells compared to the control and decreased IL-6 levels in HTR-8/SVneo cells (Supplementary Fig. S2E,F).

## Discussion

Our study shows that inhibition of autophagy significantly increases the invasiveness of JEG-3 and HTR-8/SVneo trophoblast cells, along with NF-κB activation and increased MMP-2 and MMP-9 levels, while decreasing the production of sFlt-1 and TNF-α. We also demonstrated that inhibition of NF-κB activity significantly alleviates autophagy inhibition-mediated enhancement of trophoblast invasion in these two cell lines. Collectively, our data provides evidence that autophagy is one causal mechanism regulating trophoblast invasiveness during pregnancy, in which the NF-κB signaling pathway and MMP-2, MMP-9, sFlt-1, and TNF-α levels are affected.

The role of autophagy in cellular invasion varies depending on the cell type and experimental conditions^27,28^. In glioblastoma and hepatocellular cancer cell lines, inhibiting autophagy reduces cellular invasion^29,30^. In contrast, in breast cancer cells and tongue squamous cell carcinoma cells, increased autophagic activity is associated with decreased tumor invasion^31,32^. In trophoblastic cells, several studies have reported conflicting results on the role of autophagy in trophoblast invasion. According to a review by Saito et al*.* autophagy inhibition by transfection of Atg4B^C74A^ or soluble endoglin under hypoxic conditions was associated with decreased invasion of HTR-8/SVneo cells^33^. Impaired autophagy in EVTs in placenta beds has also been implicated in the pathophysiology of preeclampsia^19^. For example, a high amount of p62 was found in EVTs obtained from preeclampsia patients, demonstrating autophagy inhibition in preeclampsia placenta. In contrast, in this study, we found that trophoblast invasion was significantly increased upon inhibiting autophagy by lentiviral expression of shRNAs for ATG5 or beclin-1 in both JEG-3 cells and HTR-8/SVneo cells. It is noteworthy that our invasion experiments were performed in naïve conditions under normoxia with only autophagy inhibited by knockdown of autophagy-related genes, which may account for this discrepancy. Meanwhile, the inverse relationship between autophagy activity and trophoblast invasion was also demonstrated by a previous study showing that inhibition of HIF1-α using siRNA decreased trophoblast invasion and increased autophagosome formation in HTR-8/SVneo cells^34^. Indeed, our observation of increased trophoblast invasion upon autophagy inhibition is consistent with a recent study that showed 54% increased invasion by silencing ATG5 in HTR-8/SVneo cells^35^. Another recent study also indicated that excessive autophagy limited trophoblast invasion, i.e. that invasiveness of EVT was reduced by glucose oxidase, an oxidative stress inducer, and was partially reversed by treatment with 3-MA, an autophagy inhibitor, in HTR-8/SVneo cells^15^.

It is presumable that the basal levels of autophagy in physiologically moderate hypoxia conditions during the early placentation period might facilitate trophoblast invasion. In fact, several autophagic knockout models indicated the importance of autophagy in embryonic survival and normal placentation. Completely autophagy-deficient mice lacking *Atg5* finally manifested embryonic lethality^36^, and a mouse model with targeted deletion of the essential autophagy gene *Atg7* in placental tissue showed significant placental abnormalities and lower pup weight^37^. In our previous study, TNF-α increased autophagic activity in JEG-3 cells^17^. In fact, excessive inflammatory stimuli, which induce autophagy in most cells^38^, interfere with trophoblast invasion^12^, and the production and/or release of cytokines has been known to contribute to the pathophysiology of preeclampsia and FGR^39^.

NF-κB, a nuclear transcriptional regulator, is involved in various cellular functions and plays a pivotal role in innate and adaptive immune responses^20^. NF-κB activation has been reported to trigger cancer cell migration and invasion^40^. NF-κB activation also has an important role in implantation and placental development^41^. However, the exact role of NF-κB activity in human trophoblast invasion remains unresolved. In general, NF-κB signaling was known to be upstream of autophagy activation^42^. The NF-κB system acts as a transcriptional regulator for several *ATG* genes such as *BECN1*^43^. However, recent study has revealed that autophagy and the NF-κB system are interconnected. That is, autophagy itself can degrade NF-κB signaling components through multiple signaling pathways^44^. In this study, we determined the protein expression levels of p65, an NF-κB subunit known to be degraded by autophagy^21^, in autophagy-deficient-HTR-8/SVneo and -JEG3 cells, and found that autophagy inhibition enhanced NF-κB activity by affecting the levels of p65. In this study, we found that the knockdown of ATG5 or beclin-1 significantly increased the expression of phosphorylated Akt, suggesting that activation of this pathway underlies the mechanism by which NF-κB controls trophoblast cell invasion. In fact, it has been previously demonstrated that activation of the Akt pathway is involved in autophagy and trophoblast invasion in JEG-3 and HTR-8/SVneo cells^45^.

In our data, although autophagy inhibition resulted in approximately twice the levels of NF-κB activation, this was not associated with increased production of TNF-α. On the contrary, TNF-α secretion was suppressed in both trophoblast cell lines upon autophagy inhibition. This result is consistent with a previous study showing that autophagy inhibition by 3-MA treatment strongly inhibits TLR-dependent TNF-α secretion in human peripheral blood mononuclear cells^46^. In fact, several studies regarding NF-κB activity and trophoblast cell invasion have been reported and results appear to largely depend on the context^47–49^. In the physiological conditions of a normal pregnancy, NF-κB exerts its function to regulate the production of cytokines that promote EVT invasion and enable maintaining the pregnancy^50,51^. In contrast, hyperactive NF-κB activation, observed to be up to ten-fold in preeclampsia placenta^52^, is associated with increase excessive cytokine expression, inhibiting trophoblast invasion. Therefore, this indicated that NF-κB coordinates trophoblast invasion to allow sufficient invasion, while preventing over-invasion into maternal tissues^49^. It is unclear at what point NF-κB activity may cause harm or exert physiological action in establishing pregnancy. Based on our data, we suggest that the linking mechanism of increased trophoblast invasion in relation to NF-κB activation upon autophagy inhibition seems not to be accompanied by excessive inflammatory cytokine production.

Two gelatinases, MMP-2 and MMP-9, digest type IV collagen and are among well-known key players of trophoblast invasion^53,54^. Our data show that increased invasion by autophagy inhibition in trophoblast cell lines are associated with increased MMP-2 and MMP-9 levels along with NF-κB activation. Since it is well recognized that NF-κB upregulates many MMP genes^55^, we consider increased MMP-2 (or MMP-9) levels may also be one of the mechanisms by which NF-κB controls trophoblast cell invasion upon autophagy inhibition. In fact, an NF-κB binding site is present in the promotor of the MMP-9 gene^56^. NF-κB was also shown to be involved in TNF-α-induced increase of MMP-9 expression in first trimester trophoblast cells^57^.

We also found that sFlt-1, an important anti-angiogenic factor in placental development, was significantly decreased upon autophagy inhibition in both JEG-3 and HTR-8/SVneo trophoblast cells. However, of note, we found that other angiogenic factors (PlGF and VEGF) and IL-6 showed differential responses upon autophagy inhibition in the two trophoblast cell lines. These factors were increased in JEG-3 cells upon autophagy inhibition, whereas they were all significantly decreased in HTR-8/SVneo cells. In fact, several reports have demonstrated the different characteristics of the two cell lines^58–60^. Given that JEG-3 cells were derived from choriocarcinoma, whereas HTR-8/SVneo cells were immortalized from first trimester EVTs, it was also shown that although these two cell lines displayed similar hypoxic responses, the underlying mechanisms differed between the two cell lines^59^. For example, the expression of PlGF is differentially regulated in trophoblast and non-trophoblast cells by oxygen tension^61^. Basically, HTR-8/SVneo cells have higher invasiveness than JEG-3 cells and higher expression of several pro-invasive cytokines such as IL-8^60^. It was also noted that the miRNA profiles of choriocarcinoma-derived cell lines and first trimester EVT-derived HTR-8/SVneo are different^58^. In terms of autophagy, we found that JEG-3 cells have a higher basal level of LC3-II expression and are less sensitive in response to rapamycin than HTR-8/SVneo cells. We also found that JEG-3 cells are susceptible to cellular toxicity under the treatment of bafilomycin A1 during the incubation time, precluding the assessment of trophoblast invasion in our experimental conditions. In consistent with these reports, we stained JEG-3 and HTR-8/SVneo cells for vimentin (a marker for mesenchymal cells) and E-cadherin (an epithelial marker)^62^ and found that JEG-3 cells highly expressed E-cadherin but not vimentin. Whereas HTR-8/SVneo cells expressed vimentin but not E-cadherin (Supplementary Fig. S3). Despite such dissimilarities between the two cell lines, our study showed that trophoblast invasion consistently increased upon autophagy inhibition in JEG-3 cells and HTR-8/SVneo cells. Given that the use of a single cell line as a model for trophoblast cells can carry the risk of misleading to erroneous hypotheses^58^, the use of two kinds of trophoblast cell lines can be considered one of the strengths of this study.

## Methods

### Reagents

To inhibit lysosomal protease, 10 μg/mL E-64d (permeable form, Sigma, USA) dissolved in DMSO and 2.5 μg/mL pepstatin A (Sigma) in 10% acetic acid in methanol were used for 4 h. Rapamycin 1 μM (Sigma) was used to induce autophagy in invasion assays. Bafilomycin A1 0.1 μM (Sigma) was used to inhibit autophagy in invasion assays. To inhibit NF-κB activity, an NF-κB inhibitor (100 μg/mL, sc-3060, Santa Cruz, USA), which has been previously used to specifically inhibit NF-kB activity^63,64^, or the NF-κB control (sc-3061, Santa Cruz) was used.

### Cell culture

JEG-3 choriocarcinoma cells was obtained from the American Type Culture Collection (ATCC, USA) and maintained in Dulbecco’s modified Eagle medium (DMEM, Hyclone, USA) containing 10% fetal bovine serum (FBS, Young In Frontier, South Korea) and 1% penicillin/streptomycin (Gibco, USA). HTR-8/SVneo trophoblast cells were generously provided by Dr. Charles H. Graham (Queen’s University, Kingston, Canada) and were maintained in RPMI 1640 media (Gibco) supplemented with 5% FBS and 1% penicillin/streptomycin. Cells were incubated at 37 °C in a 5% CO_2_ incubator.

### Preparation and infection of lentivirus

To prepare lentivirus-expressing shRNAs, lentiviral plasmids encoding either shRNA for ATG5 (TL314610, Origene, USA) or beclin-1 (GIPZ Becn1 shRNA RHS4531-EG8678, Dharmacon, USA) or the empty vector expressing green fluorescent protein (GFP; pGFP-C-shLenti scrambled negative control plasmid, Origene, USA) with viral packaging vectors were transfected into HEK-293T cells using Lipofectamine 2000 (Invitrogen, USA). Twenty-four hours after transfection, media was changed to DMEM complete media containing 10% FBS and 1% penicillin/streptomycin. Twenty-four hours after media change, the supernatant was collected and cells were incubated in fresh complete growth media. Twenty-four hours later, the supernatant was collected and combined with the previously collected sample. The supernatant (containing lentivirus particles) was filtered using 0.45 μM syringe filters (Millipore, USA) and stored at − 80 °C in 1 mL aliquots. JEG-3 and HTR-8/SVneo cells (2 × 10^5^ per 60 mm dish) were infected with 1 mL viral supernatant with polybrene (8 μg/mL) and incubated for 6 h. Media were changed to DMEM growth media containing 2 μg/mL puromycin (Sigma Aldrich,USA), a selection marker for virus infection. Virus infected cells were grown in media containing puromycin until uninfected cells in control dish were all dead and surviving cells from puromycin treatment were used for experiments.

### Invasion assay

Before plating cells, the undersides of the inserts were coated with gelatin and the insides of the Transwell inserts (8-μm pores, Costar, USA) were coated with 100 μL/insert of Matrigel (200 –300 μg/mL, BD Bioscience, USA) using a 1-h incubation at 37 °C in a CO_2_ incubator. JEG-3 (1 × 10^5^ cells) and HTR-8/SVneo (2 × 10^4^ cells) cells in 100 μL of serum-free DMEM or RPMI containing 0.5% BSA were plated onto Transwell inserts and 600 μL of complete growth media was added into the lower chamber. Cells were incubated for 48 h in a CO_2_ incubator at 37 °C. After incubation, cells were fixed with 100% methanol and stained with hematoxylin and eosin (H&E). Non-invasive cells were removed using a cotton swab and after mounting the insert membrane, cells that had invaded were counted under a light microscope (100X, Olympus BX51). For rapamycin and bafilomycin treatment, washed cells were resuspended with serum-free media containing 0.5% BSA and each drug before seeding into the upper chamber. For NF-κB inhibition experiments, the NF-κB inhibitor was added to the serum-free media containing 0.5% BSA when cells were seeded in the upper chamber, and then cells were subjected to the same incubation and counting procedures as described above.

### NF-κB activity assay

To measure NF-κB activity, JEG-3 (1 × 10^5^ cells) and HTR-8/SVneo (5 × 10^4^ cells) cells were plated in 24-well plates. Twenty-four hours after plating, plasmids encoding NF-κB-responsive firefly luciferase (NF-κB-Luc) and *Renilla* luciferase (pRL-SV40), which was used as an internal control for transfection efficiency, were transiently transfected into cells using Lipofectamine 2000 (Invitrogen). Twenty-four hours after transfection, cells were assayed for luciferase activity using the Dual-Luciferase reporter assay kit (Promega, USA). All assays were performed in triplicate and repeated three times.

### Western blotting

JEG-3 and HTR-8/SVneo cells were lysed in RIPA buffer containing 1 mmol/L phenylmethylsulfonyl fluoride (PMSF, Sigma Aldrich), 50 mM sodium fluoride (NaF, Sigma Aldrich), 1 μg/mL aprotinin (Sigma), 1X protease inhibitor (Sigma Aldrich) and 50 mM β-glycerophosphate (Sigma Aldrich). Protein concentrations were determined using a Bio-Rad protein assay (Bio-Rad, USA). Proteins were separated by SDS–polyacrylamide gel electrophoresis under reducing conditions, and then proteins were electrotransferred to polyvinylidene difluoride membranes (Millipore,). After blocking membranes with 5% non-fat dry milk in TBST, membranes were incubated with antibodies against ATG5 (Novus, USA), Beclin-1 (Cell signaling, USA), NF-κB/p65 (Santa Cruz,), LC3 (Novus), phosphorylated Akt at serine 473 and Akt (Cell signaling), and β-actin (Santa Cruz). After several washes, blots were incubated with the appropriate secondary antibody for 1 h. After an additional wash, light development was initiated by adding chemiluminescence reagents (ECL reagent, Amersham, UK).

### MMP-2 and MMP-9 concentration measurement

To measure MMP-2 and MMP-9 levels, JEG-3 and HTR-8/SVneo stable cells expressing control shRNA or beclin-1 shRNA were plated at 2 × 10^5^ cells/well into 6-well plates. Media was collected after either 24 h (for HTR-8/SVneo cells) or 48 h incubation (for JEG-3 cells) and secreted MMP-2 and MMP-9 levels were measured with an ELISA kit (R&D systems, USA) according to the manufacturer’s protocol.

### sFlt-1, PlGF, VEGF-A, TNF-α, and IL-6 concentration measurement

The levels of sFlt-1, PlGF, VEGF-A, TNF-α, and IL-6 upon autophagy inhibition were measured by an ELISA kit (R&D systems, USA) according to the manufacturer’s protocol.

### Statistical analysis

All experiments were performed at least three times. Data are presented as the mean ± SEM. Student’s *t*-test was used for statistical analysis and statistical differences were considered significant when *p < 0.05; **p < 0.01, ***p < 0.001. The chi-square test or Mann–Whitney U test was also used to analyze the results from ex vivo tissues.

## Supplementary information

Supplementary Information 1.
