# Supplementary material for: Autophagy regulates trophoblast invasion by targeting NF-κB activity
Source: Sci Rep. 2020 Aug 20;10:14033. doi: 10.1038/s41598-020-70959-2 (PMC7441061; doi:10.1038/s41598-020-70959-2)

Supplementary Information

Autophagy regulates trophoblast invasion by targeting NF-κB activity

Soo-young Oh[a,†], Jae Ryoung Hwang[b,†], Minji Choi[a], Yoo Min Kim[a], Jung-Sun Kim[c], Yeon-Lim Suh[c], Suk-Joo Choi[a], Cheong-Rae Roh[a*]

[a] *Department of Obstetrics and Gynecology, Samsung Medical Center, Sungkyunkwan University School of Medicine, Seoul 06351, Republic of Korea*

[b] *Sungkyunkwan University School of Medicine, Samsung Biomedical Research Institute, Samsung Medical Center, Seoul 06351, Republic of Korea*

[c] *Department of Pathology, Samsung Medical Center, Sungkyunkwan University School of Medicine, Seoul 06351, Republic of Korea*

***Correspondence and requests for materials should be addressed to CRR (**crroh@skku.edu).

Supplementary Figures

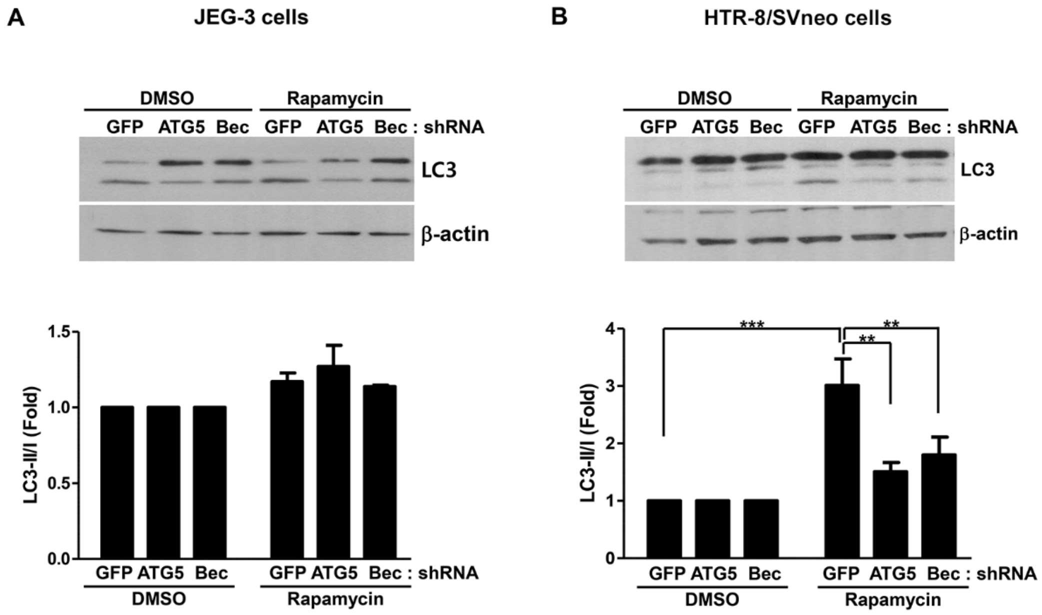

Supplementary Figure S1. Rapamycin treatment in autophagy-deficient JEG-3 and HTR-8/SVneo cells.

Rapamycin was treated to JEG-3 and HTR-8/SVneo cells upon treatment of ATG5 or beclin-1 shRNA. Cells were treated with rapamycin for 48 h. Three different experiments were performed and representative blots are shown with densitometric analysis for these experiments. The amount of LC3-II normalized to the amount of β-actin is represented by a bar graph (n=3, **, $p < 0.01$, ***, $p < 0.001$).

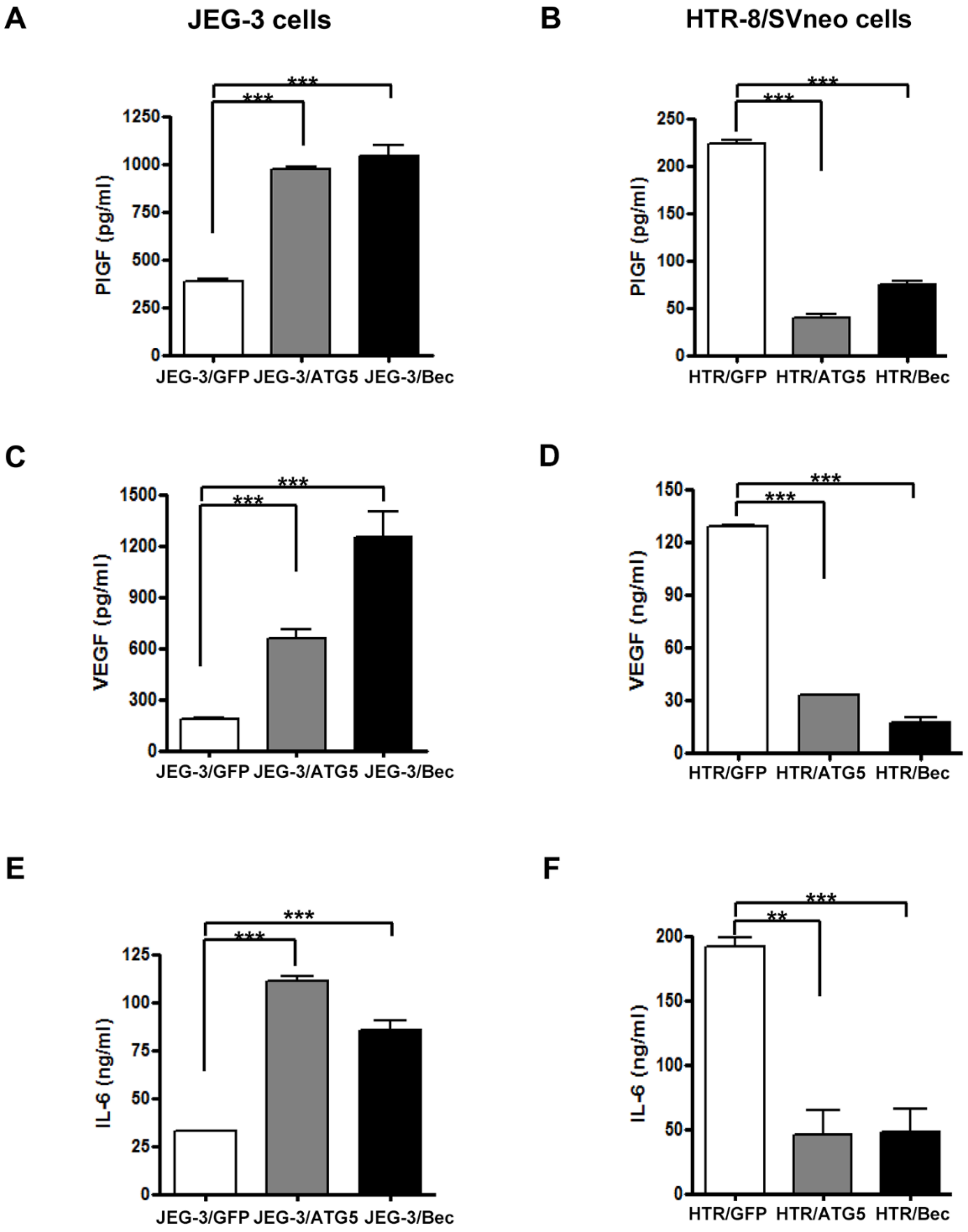

Supplementary Figure S2. The expression levels of PlGF, VEGF and IL-6 in autophagy-deficient JEG-3 and HTR-8/SVneo cells.

A and B. PlGF levels were measured by ELISA in the media obtained from JEG-3 and HTR-8/SVneo cells upon treatment of ATG5 or beclin-1 shRNA. Results are shown as the mean ± SEM of triplicate observations from three different experiments (n = 3, ***, $p < 0.001$).

C and D. VEGF levels were measured by ELISA in the media obtained from JEG-3 and HTR-8/SVneo cells upon treatment of ATG5 or beclin-1 shRNA. Results are shown as the mean ± SEM of triplicate

observations from three different experiments (n = 3, ***, $p < 0.001$).

E and F. IL-6 levels were measured by ELISA in the media obtained from JEG-3 and HTR-8/SVneo cells upon treatment of ATG5 or beclin-1 shRNA. Results are shown as the mean ± SEM of triplicate observations from three different experiments (n = 3, **, $p<0.01$, ***, $p < 0.001$).

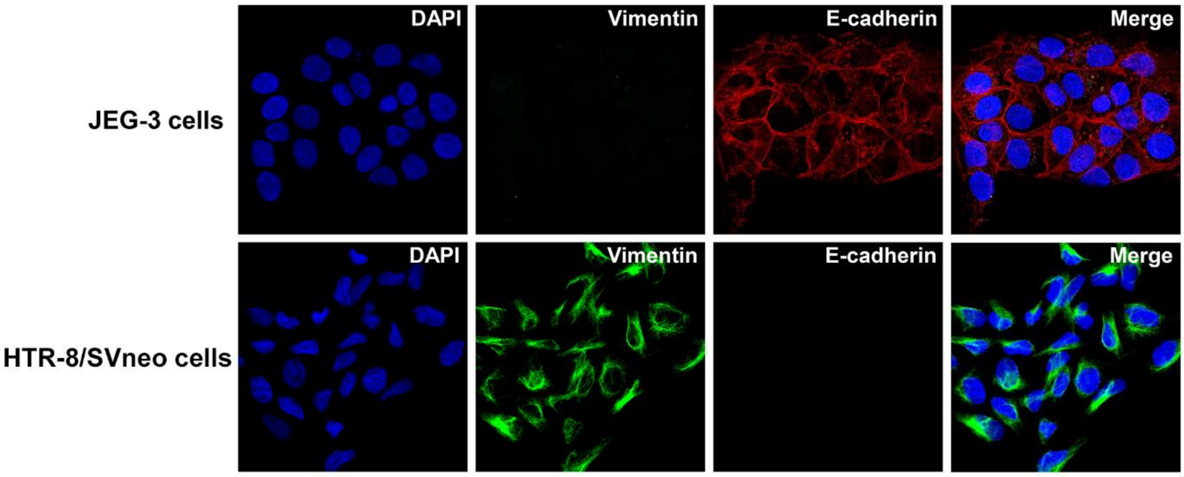

Supplementary Figure S3. Differential expression of vimentin and E-cadherin between JEG-3 and HTR-8/SVneo cells.

JEG-3 and HTR-8/SVneo cells were incubated with primary antibodies for vimentin, a mesenchymal marker and E-cadherin, and epithelial marker. Anti-rabbit IgG Alexa Fluor 488 and anti-mouse IgG Alexa Fluor 568 secondary antibodies were used for detecting vimentin and E-cadherin, respectively. DAPI (blue color) was used for staining the nucleus.

Uncropped Figures

Fig. 1A

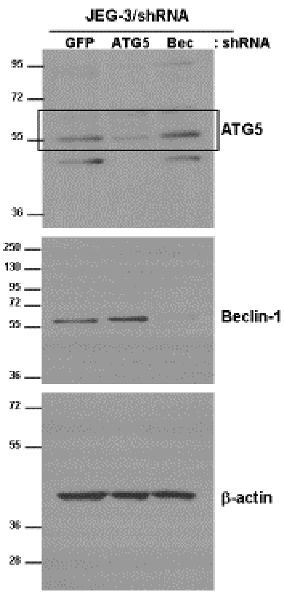

Fig. 1B

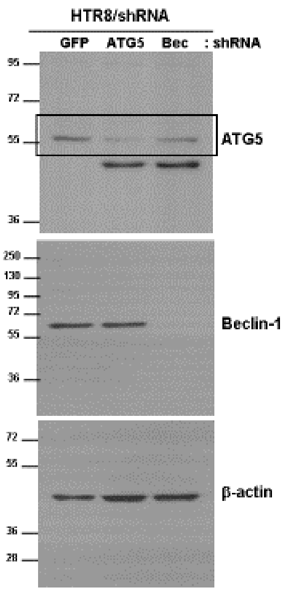

Fig. 1C

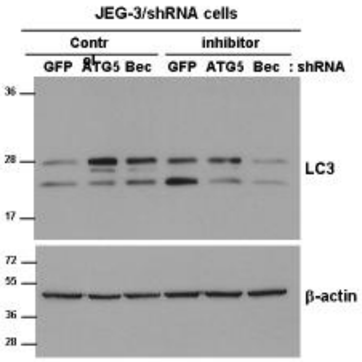

Fig. 1D

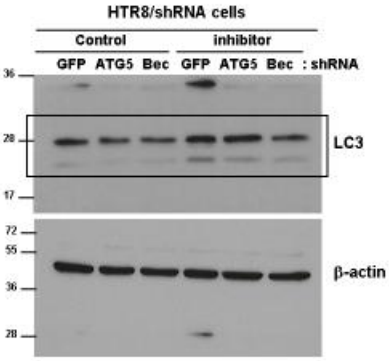

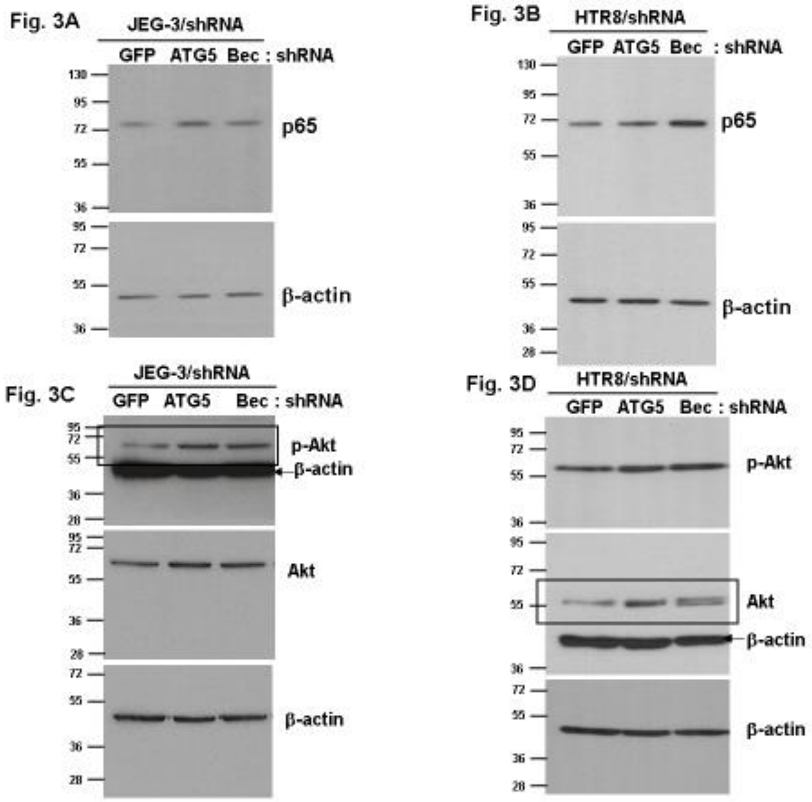

Supplementary Figure S2

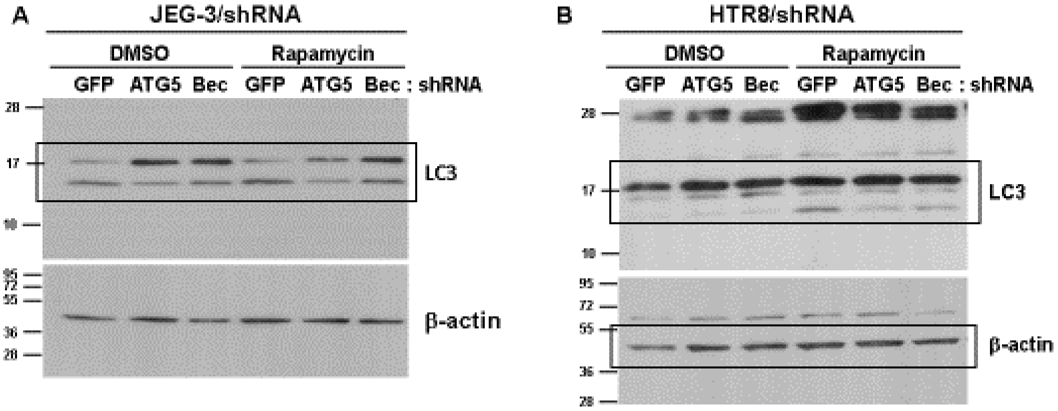

Supplement: Supplementary file 1 — Supplementary Information 1. [file 41598_2020_70959_MOESM1_ESM.pdf]
